# Autophagy Roles in Genome Maintenance

**DOI:** 10.3390/cancers12071793

**Published:** 2020-07-04

**Authors:** Susanna Ambrosio, Barbara Majello

**Affiliations:** 1 Telethon Institute of Genetics and Medicine (TIGEM), 80078 Pozzuoli, Naples, Italy; s.ambrosio@tigem.it; 2 Department of Biology, University of Naples ‘Federico II’, 80138 Naples, Italy

**Keywords:** autophagy, DNA damage response, DNA damage repair, cancer therapy

## Abstract

In recent years, a considerable correlation has emerged between autophagy and genome integrity. A range of mechanisms appear to be involved where autophagy participates in preventing genomic instability, as well as in DNA damage response and cell fate decision. These initial findings have attracted particular attention in the context of malignancy; however, the crosstalk between autophagy and DNA damage response is just beginning to be explored and key questions remain that need to be addressed, to move this area of research forward and illuminate the overall consequence of targeting this process in human therapies. Here we present current knowledge on the complex crosstalk between autophagy and genome integrity and discuss its implications for cancer cell survival and response to therapy.

## 1. Introduction

**Figure 1:**
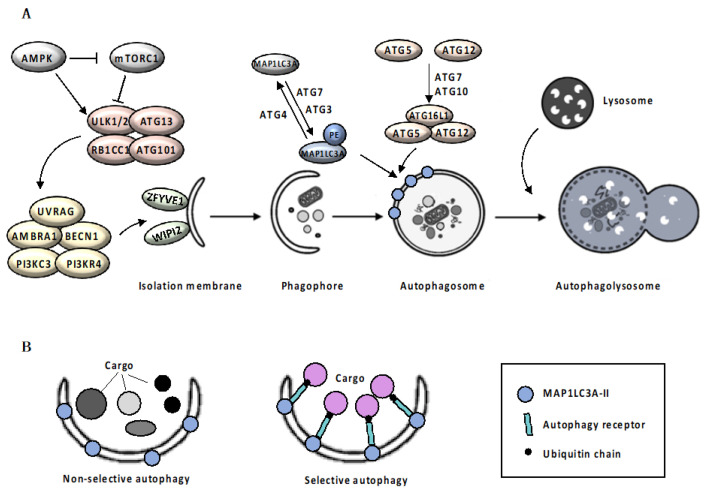
Autophagic Machinery. Box 1: Mechanism of autophagy. Autophagy initiates with the elongation of a precursor structure, called phagophore, surrounding cargo, to generate a double-membrane vesicle, called autophagosome [8,9]. Autophagosomes biogenesis is orchestrated by a subset of autophagy-related (ATG) proteins [10]. Upon autophagic stimuli, activation of AMPK (adenosine monophosphate (AMP)-activated protein kinase) and inhibition of mTORC1 (mammalian target of rapamycin complex 1) signaling pathway [11,12] leads to the activation of the ULK1/ULK2 (UNC51-like autophagy activating kinase 1/2) complex, which in turn activates the nucleation complex, including ATG13, RB1CC1 (RB1-inducible coiled-coil 1) and ATG101, which orchestrates the recruitment of further ATG proteins on the phagophore, promoting membrane elongation [13]. Nucleation complex phosphorylates AMBRA1 (autophagy and beclin 1 regulator 1) and BECN1 (beclin1), disrupting the inhibitory association with BCL2 (BCL2 apoptosis regulator) and allowing PI3KC3 (phosphatidylinositol 3-kinase catalytic subunit type 3) complex assembly [14]. The PI3KC3 complex, including the PI3KC3, PIK3R4 (PI3KC3 regulatory subunit 4), UVRAG (UV radiation resistance associated), AMBRA1 and BECN1, controls the membrane nucleation stage and initial phagophore formation [15]. PI3P (phosphatidylinositol 3-phosphate) that is generated by PI3K activity in the newly formed membranes, serves as a landing pad, known as omegasomes, for effector proteins such as ZFYVE1 (zinc finger FYVE-type containing 1) and WIPI2 (WD repeat domain phosphoinositide-interacting 2), to promote the formation of isolation membrane [16]. Elongation of the isolation membrane requires the involvement on two ubiquitin-like conjugation systems. In the first system, the protease ATG4 cleaves the precursor form of MAP1LC3A (microtubule associated protein 1 light chain 3 alpha), allowing ATG7 (E1 ubiquitin-activating enzyme) and ATG3 (E2 ubiquitin-conjugating enzyme) to catalyze the MAP1LC3A-I conjugation with phosphatidylethanolamine, to form MAP1LC3A-II [17,18]. At the same time, ATG12 is covalently conjugated to the ATG5 protein through the action of ATG7 and ATG10 (E2 ubiquitin-like enzyme) proteins [17,18]. Then, recruitment of the ATG16L1 protein (E3 ubiquitin-protein ligase) stabilizes the ATG12-ATG5 complex. ATG12-5-16L1 oligomers facilitates MAP1LC3A-II localization to the phagophore membrane, where it drives autophagosomal maturation. Once autophagosome maturation is finished, ATG4 catalyzes the reverse modification reaction of MAP1LC3A-II to MAP1LC3A-I [18]. Following completion and closure, the autophagosome ultimately undergoes fusion with a lysosome (Figure 1A). The fusion process is regulated by lysosomal membrane and cytoskeletal proteins. Finally, the fusion results in the exposure of autophagosomal cargo to the lysosomal acid hydrolases required for degradation and recycling [18]. Autophagy can be classified as non-selective or cargo-specific [19]. Selectivity of autophagy is ensured by two groups of autophagic cargo receptors, the ubiquitin-binding type, exemplified by SQSTM1 (sequestosome-1) [20] and CALCOCO2 (calcium binding and coiled-coil domain 2) [21], and the trans-membrane type, exemplified by BNIP3 (BCL2 interacting protein 3) and BNIP3L (BCL2 interacting protein 3 like) [22,23]. Although not essential for autophagosomal biogenesis itself, receptor proteins specifically bind the cargo material and the autophagosomal membrane, acting as a bridge that promotes cargo sequestration by the nascent autophagosome [24] (Figure 1B).

Autophagy is an evolutionarily highly conserved catabolic mechanism, essential to maintain normal cellular physiology [1]. Defective autophagy has been implicated in the pathology of many human diseases, such as neurodegenerative pathologies, autoimmunity, obesity, diabetes and cancer [2,3]. There are distinct types of autophagy, which are morphologically distinct based on the inducing signals, type of cargo and mechanism of sequestration: macroautophagy, microautophagy and chaperone-mediated autophagy (CMA) [4]. Macroautophagy (herein after called autophagy), mediates the sequestration of cytoplasmic material in a double-membrane vacuole and delivers it to the lysosome for degradation (see Figure 1 and Box 1 for details).

In normal conditions, autophagy operates at a low basal level to ensure the turnover of wasted organelles and macromolecules, through their lysosomal degradation and recycling, and maintains cellular metabolic homeostasis [5]. However, the autophagic flux can be upregulated in response to several stimuli, including nutrient starvation, oxidative stress, infection, protein aggregates and endoplasmic reticulum (ER) stress, to ensure stress adaptation and promote cell survival.

Despite being a cytoplasmic process, autophagy actively participates in maintaining genomic integrity, as well as in repair processes and cell fate decision after DNA damage. Impairment of autophagy has been linked to increased susceptibility to genotoxic agents and autophagy-deficient cells display more unstable genomes than cells with functional autophagy [6]; moreover, autophagic activity has been found to decrease with age, likely contributing to a reduction in efficiency of DNA repair in aged cells and tissues [7]. We then focus on recent advances in our knowledge of the reciprocal regulation connecting autophagy, genome maintenance and DNA repair.

## 2. DNA Damage Response

The genome in each of our cells can experience thousands of potentially toxic lesions from exogenous and endogenous sources every day, but highly competent mechanisms operate in concert to maintain genome integrity. Accumulation of somatic and germline mutations is at the core of oncogenesis, and deficiencies in genes responsible for efficacy of DNA repair processes have been shown to strongly increase cancer risk [25]. 

Genome integrity and repair are controlled by multiple signaling pathways. DNA repair processes are activated in the DNA damage response (DDR) and involve different biochemical pathways that can restore DNA integrity. Sensing DNA damage results in the initiation of an organized sequence of events, including DNA damage removal, signal transduction, chromatin rearrangement, damage tolerance processes, cell cycle arrest and, finally, in case of any unrepaired or excessive DNA damage, induction of cellular senescence or cell death [26]. 

In mammalian cells, the signaling cascade is initiated by members of PIKK (phosphatidylinositol 3-kinase (PI3K)-like kinase) family, ATM (ATM serine/protein kinase), ATR (ATR serine/threonine kinase), and DNA-PKcs (DNA-dependent protein kinase catalytic subunit) [27]. One of the first factors recruited to DSBs is the MRN complex, composed by MRE11 (MRE11 homolog, double strand break repair nuclease), RAD50 (RAD50 double strand break repair protein) and NBN (nibrin) [28]. The MRN complex plays critical roles in initiation of the signaling pathway, including the rapid activation and localization of ATM and other upstream signaling molecules [28]. Upon their recruitment to DNA damage sites, ATM and ATR target and activate CHEK1 (serine/threonine checkpoint kinase 1) and CHEK2 (checkpoint kinase 2), which transduce DNA damage signals to the G_1_-S, intra-S and G_2_-M checkpoints of the cell cycle [29,30]. 

Chromatin structure represents an integral part of DDR and is remodeled in response to DNA damage. Specific histone marks on the chromatin flanking DSBs, such as the phosphorylation of the histone H2A variant H2AX and the conjugation of ubiquitin chains to histone H2A [31], serve as a platform for recruitment of additional DDR factors, promoting DSB repair and resulting in amplification of signaling pathways [31]. 

While initial signaling events are immediately achieved and propagated by posttranslational modifications, an additional important component of DDR is the slower transcriptional response, that allows integration of information over time. The transcription factor TP53 (tumor protein p53) is the most extensively studied component of DDR and modulates the expression of genes involved in regulation and progression through the cell cycle, apoptosis or senescence in response to DNA damage [32,33]. 

**Figure 2:**
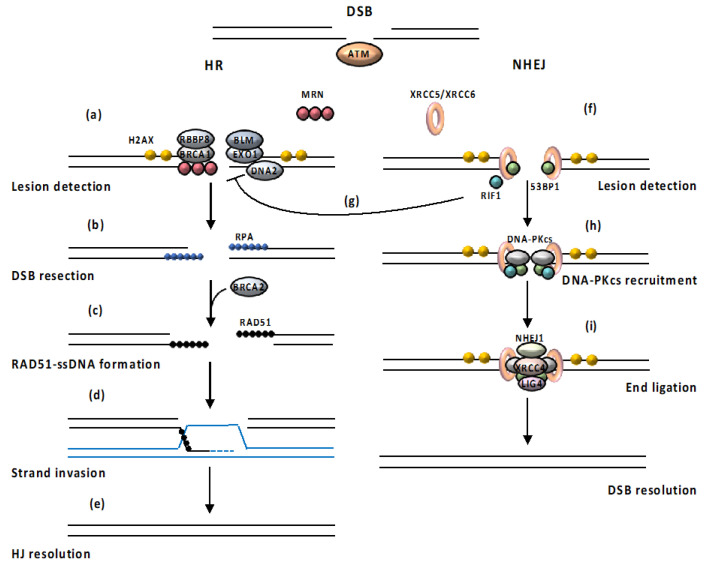
Mechanisms of DSB repair. (a) Following the recruitment and activation of ATM (ATM serine/protein kinase), the nuclease MRN (MRE11 (MRE11 homolog, double strand break repair nuclease)/RAD50 (RAD50 double strand break repair protein)/NBN (nibrin), along with its cofactor RBBP8 (RB binding protein 8) and other accessory proteins, such as BRCA1(BRCA1 DNA repair associated), initiates the 5′-3′ resection of the DNA ends, resulting in the creation of 3′-OH ssDNA tails on both ends of the break. Next, long range resection is achieved by EXO1 (exonuclease 1), DNA2 (DNA replication helicase/nuclease 2) and BLM (BLM RecQ like helicase). (b) The ssDNA-ends are coated and stabilized by RPA (replication protein A). (c) RPA is replaced from ssDNA ends by RAD51 (RAD51 recombinase) in a BRCA2 (BRCA2 DNA repair associated)-dependent process and formation of the RAD51 multimeric filament on ssDNA occurs. (d) RAD51-ssDNA filament can then strand invade a homologous dsDNA, leading to displacement on one strand of the intact DNA duplex and formation of a D-loop. (e) Following strand exchange, the resulting Holliday junction is resolved. (f) NHEJ (Non-homologous end joining) pathway initiates with the ATM-mediated phosphorylation of 53BP1, required for the recruitment of the anti-resection proteins RIF1 (replication timing regulatory factor 1) to the DSBs (double strand breaks) and inhibition of DNA resection (g). 53BP1 (TP53 (tumor protein p53)-binding protein 1) promotes the binding of XRCC5/XRCC6 (X-ray repair cross-complementing protein 6 and 5) heterodimer to the two ends of the DSB. (h) DNA-PKcs (DNA-dependent protein kinase catalytic subunit) acts as a scaffold protein that tethers the broken ends together. (i) The LIG4 (DNA ligase 4)/XRCC4 (X-ray repair cross complementing 4)/NHEJ1 (non-homologous end joining factor 1) complex ligates the DNA ends. Black arrows (↑) and perpendicular lines (⊥) indicate activation and repression, respectively.

Depending on the type of the DNA damage, cells activate different DNA repair pathways, which are specific for each type of lesion. Double strand breaks (DSBs) are primarily removed by homologous recombination (HR) and non-homologous end joining (NHEJ) DNA damage repair mechanisms [26,34] (Figure 2). 

HR is active only during the S and G2 phases, when sister chromatids are available as templates for repair [26,34]. Following the recruitment and activation of one of the damage sensor proteins, such as ATM kinase, the nuclease MRN, along with its cofactor RBBP8 (RB binding protein 8) and other accessory proteins such as BRCA1 (BRCA1 DNA repair associated), initiates the 5′-3′ resection of the DNA ends, resulting in the creation of 3′-OH single- stranded DNA (ssDNA) tails on both ends of the break [35,36]. Next, long range resection is achieved by two alternate pathways involving EXO1 (exonuclease 1) alone or BLM (BLM RecQ like helicase) working in conjunction with EXO1 or DNA2 (DNA replication helicase/nuclease 2) [37]. Afterwards, the ssDNA-ends are coated and stabilized by RPA (replication protein A), a heterotrimeric complex [38]. In the next step, RPA is replaced from ssDNA ends by RAD51 (RAD51 recombinase) in a BRCA2 (BRCA2 DNA repair associated)-dependent process [39]. After that, formation of the RAD51 multimeric filament on ssDNA occurs; RAD51-ssDNA filament can then strand invade a homologous dsDNA, leading to displacement on one strand of the intact DNA duplex and formation of a D-loop, required for prime DNA synthesis by the 3´ OH of the invading strand, in order to restore the integrity at the break point [26,39]. Finally, strand exchange occurs and the resulting Holliday junction is resolved (HJ) [34]. However, HR can also proceed by break-induced replication and synthesis-dependent strand annealing [40]. The use of sister chromatids as templates for repair by HR allows error-free repair. By contrast, the NHEJ pathway relies on the ligation of DNA ends without the use of a template, resulting in a faster, but potentially inaccurate re-ligation of DSBs [41]. The NHEJ pathway initiates with the ATM-mediated phosphorylation of the N-terminal domain of the signaling factor 53BP1 (TP53-binding protein 1), which is required for the recruitment of the anti-resection proteins RIF1 (replication timing regulatory factor 1) and PAXIP1 (PAX-interacting protein 1) to the DSBs [41]. 53BP1 then promotes NHEJ, initiated by the binding of a heterodimer, consisting of XRCC6 and XRCC5 (X-ray repair cross-complementing protein 6 and 5), to the two ends of the DSB and the interaction with the catalytic subunit of DNA-PKcs [42]. DNA-PKcs acts as a scaffold protein that tethers the broken ends together. A cascade of protein phosphorylation reactions activates and recruits other NHEJ component proteins, resulting in the excision of single-stranded overhangs of broken ends. Ultimately, the LIG4 (DNA ligase 4)/XRCC4 (X-ray repair cross complementing 4)/NHEJ1 (non-homologous end joining factor 1) complex ligates the DNA ends [43,44]. The recruitment of 53BP1 to specific histone marks is thought to tip the balance in favor of NHEJ [45,46]. The establishment of the 53BP1 barrier inhibits DNA resection, the critical step in HR, allowing NHEJ to be the first-choice pathway even in the S/G2 phase. However, if NHEJ does not occur, repair switches to HR through the RBBP8-dependent stimulation of MRE11 endonuclease activity [46,47]. 

## 3. Modulation of Autophagy by DNA Damage Response

**Figure 3:**
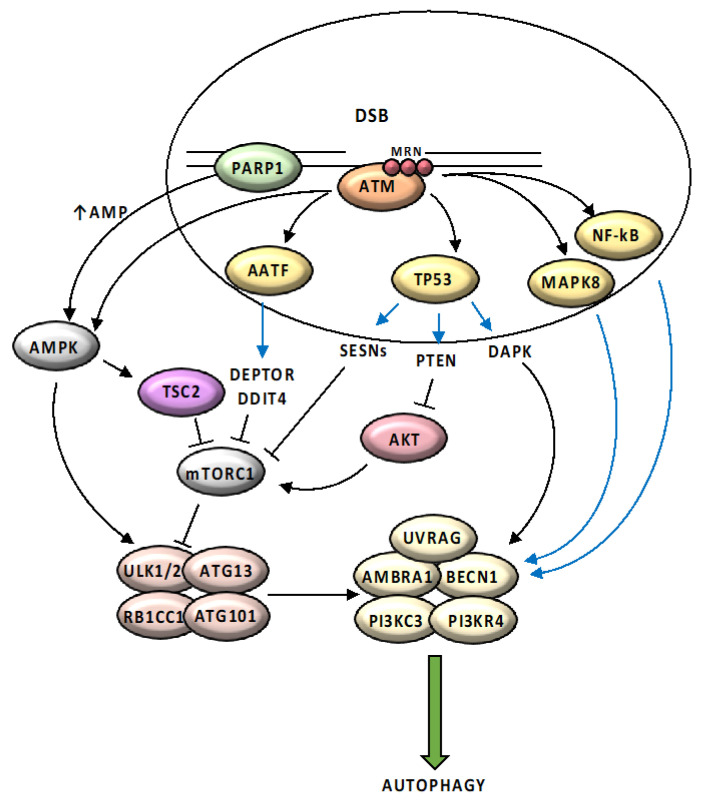
Schematic representation of main pathways regulating the DDR-mediated autophagic response. ATM is activated in response to DNA damage by the MRN complex and initiates a pathway that results in activation of AMPK and its target TSC2 (TSC complex subunit 2), which removes the inhibitory effect of MTORC1 on autophagy, promoting ULK1-dependent autophagosome formation. ATM directly phosphorylates and stabilizes TP53, which activates the expression of several regulators of the autophagic pathway including SESNs (Sestrin family genes), DAPK (death-associated protein kinase 1) and PTEN (phosphatase and tensin homolog). ATM contributes to the activation of AATF (apoptosis antagonizing transcription factor) and leads to increased transcription of two mTOR inhibitors, *DDIT4* (DNA damage inducible transcript 4) and DEPTOR (DEP domain containing MTOR interacting protein). DNA damage response activates MAPK8 (mitogen-activated protein kinase 8) and NFκB (nuclear factor kappa-light-chain-enhancer of activated B cells) pathways, that induce expression of several autophagy related genes, including *BECN1*. PARP1 activation in response to DNA damage causes reduction in NAD+ (nicotinamide adenine dinucleotide) and ATP (adenosine triphosphate) pool depletion; elevated AMP (adenosine monophosphate) levels are sensed by AMPK, leading to its activation and induction of autophagy. Black arrows (↑) and perpendicular lines (⊥) indicate activation and repression, respectively. Blue arrows indicate transcriptional regulation.

Accumulating evidence suggests that autophagy is stimulated in cells that experienced diverse DNA damaging factors including radiation, chemicals, reactive oxygen species (ROS) and oncogenes, independently of the cell-cycle position [6]. Upon exposure to genotoxic conditions, rapid DNA damage response is generally followed by a delayed and protracted autophagic induction that relies on the activation of multiple, specific pathways. Stress-responsive factors including ATM, TP53 and PARP1 (poly (ADP-ribose) polymerase 1), have been shown to play a major role in the regulation of the DDR-mediated autophagic response (Figure 3). 

ATM is activated in response to DNA damage by the MRN complex and has been described as an important link between the DDR and the induction of autophagy [28]. Autophosphorylation of ATM after DNA damage causes the activation of STK11 (serine/threonine kinase 11)/AMPK pathway, which in turn phosphorylates TSC2 (TSC complex subunit 2) and removes the inhibitory effect of MTORC1 on autophagy, promoting ULK1-dependent autophagosome formation [48,49,50].

In addition, ATM directly phosphorylates and stabilizes TP53 [32]. According to its subcellular localization, TP53 is involved in the control of autophagy through two opposite mechanisms. Cytoplasmic TP53 has been associated with activation of mTOR and inhibition of autophagy, under unstressed conditions [51,52]; conversely, nuclear TP53 binds the promoter region of several autophagy-related genes, including *UVRAG*, *ATG7*, *ULK1* and *ULK2*, and activates their expression [53]. Another transcriptional target of TP53, DAPK (death-associated protein kinase 1), a Ca(2+)-calmodulin regulated kinase, triggers autophagy through two different pathways, both converging in PI3KC3 complex activation and autophagy initiation: by directly phosphorylating BECN1, thereby releasing it from its inhibitors BCL2 and BCL2L1 (BCL2 like 1), and by phosphorylating PRKD1 (protein kinase D1), which in turn phosphorylates and activates PI3KC3 [54,55]. Additionally, TP53 activity establishes the major link between autophagy and DNA damage through the transcriptional activation of several genotoxic stress responsive factors, such as DRAM1 (DNA damage regulated autophagy modulator 1), a lysosomal protein that facilitates different stages of autophagosome formation [56,57], and SESNs (Sestrin family genes), involved in cellular response to different stress conditions [58,59,60]. TP53 also inhibits mTORC1 via transcriptional activation of several mTORC1 inhibitors, such as IGFL1 (IGF like family member 1), TSC2, PTEN (phosphatase and tensin homolog) and the beta 1 subunit of AMPK [61]. Intriguingly, the majority of the autophagic genes identified as TP53 transcriptional target are induced also by the other two TP53 family members, TP63 and TP73, suggesting that the entire TP53 family cooperates directly and indirectly in the modulation of autophagy [53,62]. 

In addition to the TP53 family members, a series of DDR proteins have been demonstrated to transcriptionally regulate autophagy components. E2F1 (E2F transcription factor 1), master regulator of cell cycle progression, promotes mTORC1 activity and represses autophagy by enhancing the expression of lysosomal v-ATPase [63]. However, E2F1 can also stimulate upregulation of genes involved in autophagy in response to DNA damage [64]. Other examples are provided by MAPK8 (mitogen-activated protein kinase 8) which, in response to specific death-promoting stimuli, upregulates the expression of several autophagy-related genes, including *ATG5*, *ATG7*, *MAP1LC3A*, and *BECN1* [65,66], the NF-κB (nuclear factor kappa-light-chain-enhancer of activated B cells) complex, which positively regulates *BECN1 and SQSTM1* [67,68], and *AATF (apoptosis antagonizing transcription factor)*, a RNA polymerase II-binding protein activated by the DNA damage response, which induces the expression of two mTOR inhibitor genes, *DDIT4* (DNA damage inducible transcript 4) and *DEPTOR* (DEP domain containing MTOR interacting protein), to sustain stress-induced autophagy [69].

Another key DDR protein involved in autophagy regulation is PARP1, a NAD+ dependent chromatin-associated enzyme acting as a molecular sensor of DNA single-strand breaks (SSB) [70]. Upon binding to DNA, PARP1 catalyzes the conversion from NAD+ to polymers of poly-ADP-ribose, a post-translational modification called PARylation. When hyperactivated by genotoxic stress, PARP1 causes a reduction in NAD+ (nicotinamide adenine dinucleotide) and the ATP (adenosine triphosphate) pool depletion. Elevated AMP levels are sensed by AMPK, leading to its activation and induction of autophagy [71]. In 2016, Rodriguez-Vargas et al. showed that transient AMPK PARylation by PARP1 during starvation is an event needed to induce AMPK nuclear export and activation, and then the initiation of autophagy [72]. Another study demonstrates that PARP1 activates autophagy in cardiomyocytes via modulating *FOXO3* (Forkhead box O3) transcription. Upon starvation, PARP1 activation promoted FOXO3 nuclear accumulation and binding activity to the target promoters, resulting in increased expression of autophagy-related genes [73]. 

## 4. Modulation of DNA Damage Response by Autophagy

**Figure 4:**
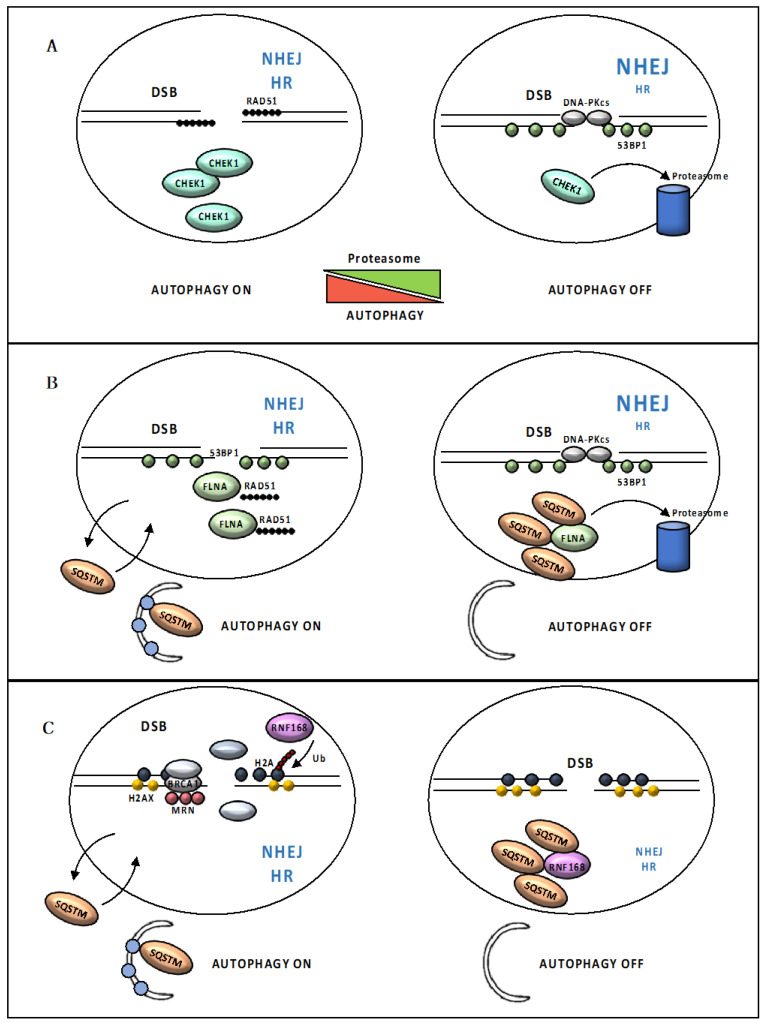
Loss of autophagy impairs DSB repair. (**A**) Autophagy indirectly regulates the CHEK1 (serine/threonine checkpoint kinase 1) levels through inhibition of its proteasome-mediated degradation. Loss of autophagy leads to deficiency in CHEK1 as a result of uncontrolled CHEK1 degradation, compromising RAD51 foci formation and causing HR deficiency. (**B**) Nuclear SQSTM1 targets FLNA (filamin A) and RAD51 for degradation via the proteasome within the nucleus, resulting in reduced levels of nuclear RAD51 and facilitating NHEJ at the expense of HR. (**C**) Increased SQSTM1 levels also suppress the E3 ligase activity of RNF168. RNF168-induced histone polyubiquitination is hampered and results in reduced recruitment of DDR proteins following DNA damage.

Autophagy, in turn, modulates several events and molecules from the extensive DDR cascade, such as cell cycle arrest, senescence, cellular death rates and also the activity of the DNA repair machinery [6,74,75]. As a degradative process, autophagy has been shown to control the levels of critical DDR-associated proteins. In yeast, acetylation modulates Sae2/RBBP8 turnover in an autophagy-mediated manner [76]. After DSB formation, the MRX (MRN, in human) complex forms at DSB ends. Two HDACs, Rpd3 and Hda1, keep Sae2 in the deacetylated form that influences Mre11/MRE11 dynamics at the DSB site. Once resection has taken place, Sae2 undergoes Gcn5/SAGA-dependent acetylation, that promotes Sae2 export from the nucleus and autophagy-mediated degradation, probably to counteract extensive DSB resection [76]. In mammals, control of CHEK1 levels via CMA, a selective autophagy subtype, has been proposed to positively control HR activity [77,78]. Specifically, CMA tightly regulates CHEK1 levels to prevent the MRN complex hyperphosphorylation and destabilization. Thus, CHEK1 abnormally accumulates in cells with defective CMA, compromising cell cycle progression and causing HR deficiency [77]. Autophagy has also been shown to indirectly regulate CHEK1 levels through inhibition of its proteasome-mediated degradation. Therefore, in this case, loss of autophagy leads to deficiency in CHEK1 as a result of uncontrolled CHEK1 degradation [78] (Figure 4A). RAD6, an E2 ubiquitin-conjugating enzyme that plays a pivotal role in repairing UV-induced DNA damage, has been proposed to promote the HR-repair through the ubiquitination and consequent autophagy-mediated degradation of the heterochromatin protein CBX5 (chromobox 5), involved in the formation of RAD51 nucleoprotein filaments [79]. Moreover, autophagy has been reported to be directly involved in DSB repair by regulating protein levels of the autophagosome cargo SQSTM1, specifically in the nucleus [80,81]. Nuclear SQSTM1 has been shown to dynamically associate with DNA damage foci and interact with FLNA (filamin A), which was previously implicated as a HR-regulatory protein. SQSTM1 targets FLNA and RAD51 for degradation via the proteasome within the nucleus, resulting in reduced levels of nuclear RAD51 and facilitating NHEJ at the expense of HR [80] (Figure 4B). Increased levels of SQSTM1 also suppress RNF168 (E3 ligase activity of ring finger protein 168) by directly binding to its MU1 domain [81]. This interaction hampers the RNF168-induced histone polyubiquitination, leading to reduced recruitment of DDR proteins following DNA damage. Therefore, increased SQSTM1 levels seem to be responsible for reduced DNA damage repair upon autophagy inhibition [81] (Figure 4C). Importantly, because RNF168 acts upstream of both HR and NHEJ, SQSTM1-mediated inhibition of chromatin ubiquitination perturbs both repair pathways [31,81]; in contrast, autophagy inhibition only suppresses HR, suggesting that when the whole process of autophagy is impaired, other mechanisms may rescue the SQSTM1-mediated reduction in NHEJ [81].

Overall, loss of autophagy appears to result in multiple layers of regulation that combine or counterbalance each other determining different functional consequences on DNA repair depending, apparently, on cellular context and stress-specific components. It is not surprising that an increasing body of evidence highlights the multifaceted impact of autophagy on DNA repair, in normal and cancer cells. For instance, the lack of autophagy has been shown to lead to down-regulation of proteins critical for HR, and also for NHEJ, in hematopoietic cells [82]. On the other hand, down-regulation of important autophagy players changes the percentage of irradiated cells with nuclear foci positive for 53BP1, an NHEJ marker, but not RAD51, an HR marker, in colorectal cancer cells [83]. Autophagy inhibition negatively regulates repair by NHEJ in PTEN-deficient, but not in PTEN-proficient, prostate adenocarcinoma cells [84]. Further work is required to identify precisely the mechanisms allowing autophagy to promote DNA repair and what factors determine these apparently different functions of autophagy. 

## 5. Autophagy and the DNA Excision Repair Pathways

The DNA excision repair mechanisms play a crucial role in the repair of single nucleotide defect, base loss and single strand breaks. Three pathways are involved in protection of single-strand integrity: base excision repair (BER), mismatch repair (MMR) and nucleotide excision repair (NER).

BER is the predominant DNA damage repair pathway for the processing of small base lesions that do not significantly distort the DNA helix structure, derived from oxidation and alkylation damages. BER is initiated by damage-specific DNA glycosylases, such as OGG1 (8-oxoguanine DNA glycosylase), that recognize and remove the damaged base, leaving an apurinic/apyrimidinic (AP) site which is then cleaved by an AP endonuclease, such as APEX1 (apurinic/apyrimidinic endodeoxyribonuclease 1). The AP site is subsequently processed by at least two BER sub-pathways: ‘short-patch’ BER (where a single nucleotide is replaced) and ‘long-patch’ BER (where two or more nucleotides are replaced). In the ‘short-patch’ sub-pathway, the AP site is filled by the DNA polymerase β, and the LIG3-XRCC1 (DNA ligase 3- X-ray repair cross complementing 1) complex seals the remaining nick in the phosphodiester backbone. In the ‘long-patch’ sub-pathway, a DNA polymerase (β, δ, or ε) adds 2 to 15 more nucleotides into the single-nucleotide gap, creating a flap structure which is recognized and excised by FEN1 (flap structure-specific endonuclease 1) in association with PCNA (proliferating cell nuclear antigen). Finally, LIG1 (DNA ligase 1) repairs the remaining nick [85]. 

Although the crosstalk between BER and autophagy is still poorly investigated, studies indicate a link between the OGG1 glycosylase and autophagy, under certain stress conditions. In cardiomyocytes, nutrient deprivation leads to impaired BER, due to autophagy-dependent OGG1 degradation. However, though necessary, autophagy activation itself is not sufficient for OGG1 lost, suggesting that other signaling pathways are involved [86]. On the other hand, OGG1 has been implicated in the activation of autophagy pathways in hyperoxic-stressed pulmonary cells. In this study, Ye et al. reported that, under hyperoxia, *ogg-1* knock out mice displayed decreased Atg7 and Map1lc3b-II levels and impaired autophagy [87]. 

MMR is a post-replicative repair pathway that primarily functions to remove small insertion/deletions loops (IDLs) and base-base mismatches, due to errors in replication or homologous recombination processes. MMR is initiated by the MutSα complex (MSH2–MSH6 heterodimer), which recognizes the mispair; in cases of IDLs with two or more extra bases, MutSβ (MSH2-MSH3 heterodimer) are responsible for the detection. Binding of MutSα to the single base mismatch leads to the recruitment of downstream DNA repair proteins, including MutL homologs (MLH1-PMS2 heterodimer), containing endonuclease activity, and PCNA. Excision by EXO1 leads to the formation of an RPA-coated single-strand gap; then, a DNA polymerase synthetizes the new strand to fill the gap [88]. 

MMR also mediates cytotoxicity of several classes of clinically active chemotherapy drugs including 6-thioguanine (6-TG) and 5-fluorouracil (5-FU). In response to these DNA-damaging agents, cells activate cell cycle checkpoints and programmed cell death with both apoptotic and autophagic features [89]. MMR has been implicated in activation of autophagy by 6-TG and 5-FU. Zeng et al. proposed that the MMR requires BNIP3 to activate autophagy in response to 5-FU and 6-TG treatment, in an MTOR- and TP53-dependent process [89,90,91]. Studies in *Caenorhabditis elegans* and human cells suggest that a crosstalk between the BER and MMR pathways exists for the activation of autophagy in response to 5-FU, where the MMR proteins MSH2 and MSH6 act as sensors of DNA damage induced by5-FU, and the BER proteins APN-1 and EXO3 act downstream in the same pathway, to generate a nick required for MMR activation and induction of DDR, leading to 5-FU toxicity and induction of cell death by autophagy. Importantly, activation of autophagy acts as a determinant for sensitivity to 5-FU in *C. elegans* embryos, and failure to induce autophagy correlated with drug-resistance [92].

NER repairs lesions which consist of bulky, helix-distorting damage, such as UV-induced pyrimidine dimers. Two distinct damage detection sub-pathways exist: global genomic NER (GG-NER) can occur anywhere in the genome and is initiated by the recognition of DNA helix distortions by the GG-NER-specific XPC-RAD23B complex, whereas transcription-coupled NER (TC-NER) specifically repairs lesions in transcribed strands of active genes, is initiated by RNA polymerase stalled at a lesion and depends on recruitment of TC-NER-specific factors ERCC8 (ERCC excision repair 8, CSA ubiquitin ligase complex subunit) and ERCC6 (ERCC excision repair 6, CSB chromatin remodeling factor) to the site of damage [93]. Despite different beginnings, both pathways require the recruitment of the helicase GTF2H1 (general transcription factor IIH subunit 1), that unfolds the DNA helix creating a bubble of approximately 30 base pairs and allowing the recruitment of the XPA and the RPA complex. In the next step, the endonucleases XPF/ERCC1 (ERCC excision repair 1) and XPG make the incision in damaged DNA strands; a DNA polymerase fills the gap and finally LIG1 and LIG2 perform the sequential last step of sealing the nicks [93].

Autophagy is required for efficient repair of UVB-induced DNA damage by NER. Mechanistically, autophagy deficiency induced TWIST1 (twist family bHLH transcription factor 1) stabilization and accumulation, which in turn downregulates XPC expression through the activation of the transcription repressor complex E2F4-RBL2; moreover, TWIST1 blocks the recruitment of the NER protein DDB2 (damage-specific DNA binding protein 2) to UV-induced DNA damage sites by suppressing EP300 (E1A binding protein p300) activity [94]. 

On the other side, the NER protein XPA has been proven to promote cell protective autophagy in melanoma cells treated with cisplatin, a chemotherapeutic drug. XPA promotes autophagy in resistant melanoma cells after cisplatin treatment through facilitating PARP1 activation. Inhibition of XPA-PARP1-mediated autophagy makes resistant melanoma cells more susceptible to cisplatin treatment, causing cell death through the apoptotic pathway [95].

## 6. Autophagy and Replication Stress

Although the exact mechanism behind this connection remains a matter of debate, the majority of studies provided evidence that loss of autophagy causes HR deficiency [77,80,81]. Beyond its critical role in repair of DSBs, HR deficiency in cells lacking autophagy could lead to additional vulnerabilities. For example, HR plays a major role in the resolution of replication stress in S phase [96]. Replication stress is referred to as DNA damage generated by errors during DNA replication, endogenously, e.g., by activation of oncogenes, or exogenously, e.g., by genotoxic therapies, and affects genomic loci that are particularly difficult to replicate, where replication forks could stall or collapse [97]. Under conditions of replication stress, activation of DDR coordinates cell cycle checkpoint activation and replication fork stabilization, required to prevent premature entry into mitosis with unreplicated DNA forming abnormal DNA structures, which can be transmitted to daughter cells, resulting in genome instability [97].

In a recent study [98], Vanzo and coworkers elucidated the relationship of autophagy and proper progression of the DNA replicative fork. They found that oncogene- or drugs-induced replication stress triggered DDR, which is followed by a delayed and protracted autophagic induction [98]. Importantly, autophagy gene knockout caused replication stress, but, contrary to expectation, had no detrimental effect on basal metabolism, mitochondrial function and ROS levels; moreover, normal DNA synthesis and timely recovery from replication stress required functional autophagy [98]. Unexpectedly, the authors proposed an essential role for autophagy in promoting proper DNA replication and ensuring genomic stability, by stabilizing nucleotide pools. Consistently, in autophagy deficient-cells, replication stress could be partly mitigated by exogenous supply of deoxynucleosides. Moreover, autophagy was enhanced in both early and advanced stages of human urinary bladder and prostate cancer, supporting a role for autophagy in sustaining cancer cells proliferation by supplying nucleotides for DNA synthesis [98].

Cells employ two convergent pathways for nucleotide synthesis: the de novo pathway, which synthesizes nucleotides from sugar and amino acids, and the recycling nucleoside salvage pathway, in which nucleotides are synthesized from intermediates that are formed during the degradative process of nucleic acids metabolism [99]. The intracellular levels of dNTPs are regulated by RNR (ribonucleotide reductase), which catalyzes de novo biosynthesis of deoxyribonucleotides from ribonucleotides. Consequently, RNR constitutes a regulatory factor in the total rate of DNA synthesis [100]. In yeast models, upon methyl methane sulfonate treatment, DNA damage-induced autophagy specifically targets the RNR subunit Rnr1 for degradation [101]. Reduction in Rnr1 levels favors the formation of the more efficient Rnr1–Rnr3 complex, instead of Rnr1–Rnr1, thus resulting in optimization of RNR activity and promptly promoting dNTP formation under DNA damage conditions. However, it remains unknown whether this mechanism, uncovered in yeast, also exists in mammalian cells. A critical role for autophagy in preventing nucleotide pool depletion during starvation has previously been demonstrated [102,103], while, in *C. elegans*, autophagy-dependent ribosomal RNA degradation has been shown to be essential for maintaining nucleotide homeostasis during animal development [104]. Thus, autophagy could participate in the turnover of key proteins involved in dNTP neosynthesis and/or provides metabolic substrates for salvage, through degradation of RNA and ribosomes, to maintain nucleotide homeostasis. 

## 7. Autophagy and Oxidative Stress 

**Figure 5:**
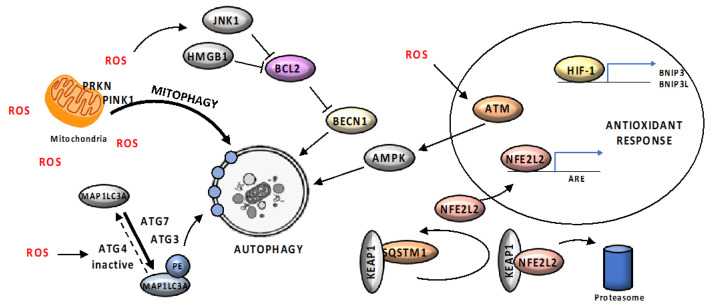
Schematic representation of main pathways linking oxidative stress to autophagy. Mitochondria, the main source of cellular ROS; in order to limit ROS damage, autophagy clears damaged and dysfunctional mitochondria by mitophagy. ROS-mediated ATG4 oxidation causes MAP1LC3A-II accumulation, promoting its association with the autophagosome membrane. Accumulation of ROS activates autophagy by inhibiting mTORC1 activity via the ATM/STK11/AMPK axis. ROS promote HMGB1 (high mobility group box 1) translocation from the nucleus to the cytosol, where it induces autophagy disrupting the inhibitory interaction of BCL2 with BECN1. In response to oxidative stress, JNK1 (c-Jun-N-terminal kinase 1) becomes activated and in turn induces autophagy by phosphorylating BCL2, thereby disrupting the BCL2/BECN1 interaction. SQSTM1 directly interacts and sequesters KEAP1 into autophagosomes, disrupting the KEAP1-mediated NFE2L2 (nuclear factor, erythroid 2 like 2) ubiquitination and leading to NFE2L2 release into the nucleus and thereby activation of specific transcription program. During ROS-induces Hypoxia, HIF-1 (Hypoxia-inducible factor 1) activate the expression of autophagy key proteins, including mitophagy receptors BNIP3 and BNIP3L, to mitigate hypoxic condition and mitochondrial ROS generation. Black arrows (↑) and perpendicular lines (⊥) indicate activation and repression, respectively. Blue arrows indicate transcriptional regulation.

Excessive ROS production may result in significant irreversible damage to organelles, alteration of membranes and DNA damage, due to their ability to react with and oxidaze biomolecules [105]. Cumulatively, these events are known as oxidative stress and are involved in mutations, cancer and many other diseases. Under physiological conditions, low levels of ROS are involved in cellular signal regulation. ROS level is regulated by antioxidant enzymes superoxide dismutases, catalase and glutathione peroxidases, which catalyze transformation of reactive oxygen species into stable non-toxic molecules and restore the reduced protein and lipid pool, therefore representing the most important defense against oxidative stress [106]. When ROS are produced at an extent high enough to overcome the antioxidant defense, autophagy becomes a crucial response to oxidative stress, by removing irreversibly oxidized proteins and organelles (Figure 5). 

Mitochondria are the main source of cellular ROS as a by-product of respiration and a tight control of mitochondria number and functioning is essential for cellular homeostasis [105,106]. In order to limit ROS damage, autophagy clears damaged and dysfunctional mitochondria by their selective sequestration and lysosomal degradation, in a process named mitophagy [107]. A critical regulator of this pathway is PINK1 (serine/threonine kinase PTEN induced kinase 1) which acts as a sensor for mitochondrial damage [108]. PINK1 is physiologically imported to the inner mitochondrial membrane to be rapidly degraded by mitochondrial proteases; however, stress-induced depolarization of the mitochondrial membrane stabilizes PINK1 at the outer mitochondrial membrane, where it recruits and phosphorylates PRKN (parkin RBR E3 ubiquitin protein ligase), resulting in an increase in its Ub-ligase activity [109]. PRKN polyubiquitinates numerous outer mitochondrial membrane proteins, leading to the recruitment of autophagy adaptors such as OPTN (optineurin), CALCOCO2 and SQSTM1, which then initiate mitophagy [107].

ROS immediately accumulate upon starvation and are able to influence multiple signaling pathways involved in autophagy regulation. For instance, ATG4, an essential factor in the process of autophagosome formation, has been identified as a direct target for oxidation by H_2_O_2_ during starvation [110]; ROS-mediated ATG4 oxidation causes MAP1LC3A-II accumulation, promoting its association with the autophagosomes membrane [110]. In addition, accumulation of ROS activates autophagy by inhibiting mTORC1 activity via the ATM/STK11/AMPK axis [111,112]. Furthermore, ROS generated by cellular stress promote HMGB1 (high mobility group box 1) translocation from the nucleus to the cytosol, where it induces autophagy disrupting the inhibitory interaction of BCL2 with BECN1 [113,114]. Similarly, JNK1 (c-Jun-N-terminal kinase 1) becomes activated following the oxidation of its upstream redox-sensitive MAP3K5 (mitogen-activated protein kinase 5), and in turn induces autophagy by phosphorylating BCL2, thereby disrupting the BCL2/BECN1 interaction [115]. On the other side, ROS-dependent activation of MAPK14 (mitogen-activated protein kinase 14) during starvation limits excessive autophagy activation in order to protect cancer cells against stress-induced cell death [116].

According to the majority of the work, ROS levels increase upon oxygen deficiency, probably due to impairment in the electron transport across the mitochondrial complexes [105,106], and autophagy is induced to mitigate the hypoxic condition and mitochondrial ROS generation. HIF-1 (Hypoxia-inducible factor 1), the master regulator of hypoxia response, mediates this response by increasing the expression of autophagy key proteins, including mitophagy receptors BNIP3 and BNIP3L [117]. 

The SQSTM1-KEAP1 (kelch like ECH associated protein 1)—NFE2L2 (nuclear factor, erythroid 2 like 2) axis exemplifies the crucial link between autophagy and oxidative stress response pathways in maintaining cellular homeostasis. NFE2L2 is a major oxidative stress response transcription factor, which induces the transcription of antioxidant response element (ARE)-containing genes [118]. The E3 ubiquitin ligase KEAP1 negatively regulates NFE2L2, promoting the NFE2L2 ubiquitin-dependent proteasomal degradation, under physiological conditions. Oxidative stress disrupts the interaction between NRF2 and its repressor, leading to NFE2L2 release into the nucleus and thereby activation of a specific stress response transcription program [118]. SQSTM1 has been shown to directly interact and sequester KEAP1 into autophagosomes, disrupting the KEAP1-mediated NFE2L2 ubiquitination, therefore stabilizing NFE2L2. As a consequence, loss of autophagy leads to accumulation of SQSTM1, and the stabilization and nuclear translocation of NFE2L2 [119]. SQSTM1 has been found as a direct target for oxidation on specific cysteine residues; oxidized SQSTM1 activates pro-survival autophagy, acting as a sensor of ROS levels [120]. Moreover, the *SQSTM1* promoter contains an ARE, suggesting the existence of an important positive feedback loop linking selective autophagy and oxidative stress response [121]. 

## 8. Autophagy, Senescence and Replicative Crisis

Senescence is an irreversible form of proliferative arrest of mitotic cells, caused by diverse intracellular or extracellular stress or damage, including telomere erosion, DNA damage, mitochondrial dysfunction and oncogenic mutation, with the purpose of limiting proliferation of damaged cells, thereby preventing malignant transformation. However, with age, persistent senescence can cause accumulation of molecules, termed the senescence-associated secretory phenotype (SASP), that reinforce senescence through autocrine pathways and create a chronic inflammatory environment that may favor the establishment of aging-related pathologies, including cancer [122]. 

The first causal relationship between autophagy and senescence came from oncogene-induced senescence (OIS), where autophagy facilitates the production of SASP [123]. A membrane compartment known as the “TOR-autophagy spatial coupling compartment” (TASCC) was identified by Narita and collaborators, where autolysosomes and mTOR accumulated during RAS-induced senescence [123]. Amino acids and other metabolites are provided as a consequence of autolysosome formation and supply basic macromolecules for the synthesis of SASP components. Disruption of MTOR localization to the TASCC suppressed synthesis of two major SASP components, interleukin 6 and 8 [123]. Alternatively, other studies show that autophagy can function as an anti-senescence mechanism. The transcription factor GATA4 (GATA binding protein 4) is physiologically targeted for degradation by autophagy through interaction with SQSTM1 [124]. Irradiation or OIS activate ATM and ATR to block SQSTM-dependent autophagic degradation of GATA4, resulting in NF-κB activation and SASP induction [124].

**Figure 6:**
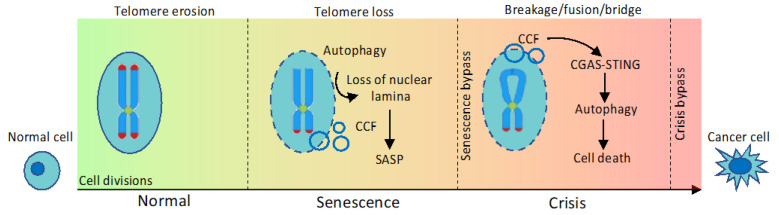
Autophagy acts as a barrier to malignant transformation. Telomeres shorten as a result of cellular replication, leading to replicative senescence. Autophagy promotes senescence through degradation of the nuclear lamina; loss of nuclear lamina leads to extrusion of fragments of chromatin and portions of nucleus, generating CCFs (cytoplasmic chromatin fragments) which are exposed to DNA-sensing pathway, promoting SASP (senescence-associated secretory phenotype). When senescence is bypassed, due to dysfunctional cell cycle check-points, cells continue to divide and could undergo breakage/fusion/bridge cycle, that involves repeated formation of chromosomal fusions and subsequent breaks initiated by the loss of telomere protection. Telomere dysfunction generates CCFs that activate the DNA-sensing CGAS–STING (cyclic GMP-AMP synthase-stimulator of interferon response cGAMP interactor 1) pathway, required for a form of cell death known as replicative crisis, preventing malignant transformation.

Autophagy also promotes senescence through degradation of the nuclear lamina [125]. In response to oncogenic activation, LMNB1 (lamin B1), a major nuclear envelope component, together with associated chromatin, is exported from the nucleus to the cytoplasm and undergoes autophagic degradation. This process is mediated by the direct interaction of MAP1LC3A with LMNB1 within the nucleus and inhibiting autophagy or the MAP1LC3A-LMNB1 interaction prevents LMNB1 loss and attenuates OIS [125]. Loss of nuclear lamina leads to the extrusion of fragments of chromatin and portions of the nucleus, generating cytoplasmic chromatin fragments (CCFs) which are exposed to the DNA-sensing pathway, promoting SASP [126] (Figure 6). 

A functional link between autophagy and the DNA-sensing pathway has also emerged recently in the context of replicative crisis [127,128]. Normal somatic cells have a finite ability to proliferate and enter senescence once they complete a certain number of replications. When senescence is bypassed, due to dysfunctional cell cycle check-points, cells continue to divide and could undergo a breakage/fusion/bridge (BFB) cycle, that involves repeated formation of chromosomal fusions and subsequent breaks initiated by the loss of telomere protection. Telomere dysfunction generates CCFs that activate the DNA-sensing CGAS–STING (cyclic GMP-AMP synthase-stimulator of interferon response cGAMP interactor 1) pathway, required for a form of cell death known as replicative crisis, preventing malignant transformation [127]. A new report by Nassour et al. provides evidence that crisis-associated cell death is dependent on autophagy [128]. Consistent with that, autophagy-deficient cells were able to escape crisis and accumulate high levels of chromosomal aberrations. Activation of autophagy depended upon chromosomal fusion and CCFs formation, as intra-chromosomal breaks failed to induce cytosolic DNA species and autophagy [128] (Figure 6). Of note, a link between CGAS–STING pathway and autophagy has been previously reported in the context of anti-microbial immune responses, where, upon cytoplasmic DNA stimulation, CGAS physically interacts with and activates BECN1, leading to autophagy-mediated degradation of cytosolic pathogen DNA [129]. 

Thus, autophagy not only stabilizes senescence, playing a role in genome maintenance, but also activates autophagic cell death at crisis, acting as a barrier to oncogenic activation at an early stage of immortalization and malignant transformation. 

## 9. Microphthalmia Family of Transcription Factors and Genome Maintenance

Lysosomes are integrally involved in the autophagic system, as they execute the degradative stage of the pathway, and impaired lysosomal activity leads to autophagic dysfunction. Thus, lysosomal number or activity must also be rapidly adapted in response to cellular need. The identification of the Microphthalmia family of transcription factors (MiT/TFE) clarified the mechanism by which cells adapt to stress for maintaining cellular and tissue homeostasis [130]. The MiT/TFE transcription factors, i.e., MITF (melanocyte inducing transcription factor), TFEB (transcription factor EB), TFE3 (transcription factor binding to IGHM enhancer 3) and TFEC (transcription factor EC), act as master regulators of intracellular clearance and energy metabolism by orchestrating the transcription of genes involved in lysosomal and autophagosome biogenesis [131]. According to current models, functions of MiT/TFE factors are regulated by phosphorylation and shuttling between the cytoplasm and the nucleus: under normal conditions, phosphorylation by mTORC1 leads to their cytoplasmic retention; conversely, upon nutrient deprivation, they are dephosphorylated and rapidly translocate into the nucleus, where they transactivate target genes [131]. Multiple studies have revealed that MiT/TFE factors are activated in a variety of cell types in response to various physiological stressors, including inflammation, ER stress and oxidative stress [131]. However, though the roles of autophagy in damage repair and genomic stability are currently well established, the transcriptional roles of MiT/TFE factors have only been poorly considered in the context of genome maintenance. 

A functional link between MiT/TFE factors and the DDR pathway has emerged recently in the context of oxidative stress [132]. Wang et al. found that ROS are able to induce a rapid nuclear translocation of TFEB, TFE3 and MITF in a mTORC1-independent manner. A common mechanism for redox-signaling is the oxidative post-translational modification of cysteine residues. Authors found that ROS-dependent TFEB, TFE3 and MITF nuclear translocation occurs due to direct oxidation of specific cysteine residues, that reduces binding affinity with RRAG GTPases, finally resulting in the induction of an autophagy-lysosome gene expression program [132].

Recently, Brady and coworkers highlighted that the MiT/TFE transcription program is integrated with DDR to maintain cell homeostasis under DNA damage [133]. They showed that TFE3 and TFEB are dephosphorylated and translocate to the nucleus in response to exposure to different genotoxic agents; TP53-dependent mTORC1 inhibition appears to be responsible for DNA damage-mediated TFE3 and TFEB activation. In turn, TFE3 and TFEB contribute to sustain TP53-dependent response by stabilizing TP53 protein levels, while TFEB/TFE3 double knock-out cells exhibit a decreased TP53 half-life and a profound dysregulation of the DNA damage response, as well as a delayed cell death in response to prolonged DNA damage [133]. Importantly, TFEB and TFE3 directly regulate expression of genes implicated in the cell cycle and proliferation, while TFEB and TFE3 genetic ablation results in reduced expression of cell cycle genes and altered cell cycle progression, which can also have relevant consequences on proper engagement of DNA repair pathways and senescence-inducing signals [133,134].

The contribution of MiT/TFE factors in maintaining genome integrity is probably complex and context dependent and further investigation in this area is needed. It remains unclear precisely which factors and mechanisms are involved in the integration of the MiT/TFE transcription program and DDR to modulate an appropriate response to different types of genotoxic insults.

## 10. Autophagy, Cancer and Response to Therapy 

As autophagy is frequently stimulated in response to many anticancer drugs, its effects on cell survival versus death have attracted particular attention in the context of malignancy, as they may affect the outcome of DNA-targeted drug treatments [135,136,137]. Autophagy is a critical player in DDR and genotoxic stress may evoke autophagy as an early adaptive pro-survival response. In this setting, autophagy has been proposed as target for cancer therapy, particularly in advanced stage cancers. There is extensive evidence for autophagy serving a cytoprotective function when challenged by antitumor drugs or radiation and the use of autophagy inhibitors combined with chemo- or radiotherapy has achieved encouraging results in several clinical trials [138,139,140,141,142,143]. However, besides its positive role in cellular survival processes, autophagy may also contribute to cell death, mediating an antitumor action. Autophagy can be important for the activation of other cell death pathways, through the selective targeting of proteins that are necessary for cell function and viability, and autophagosome membranes can act as scaffolds for cell-death signaling. Moreover, lethality may result from overconsumption of mitochondria, intracellular membranes and cytoplasmic material. This supports the idea that excessive degradation of cytoplasmic components due to extreme levels of autophagy leads to an autophagy-dependent cell death, although it remains somewhat controversial, at least in mammals [136,137]. In this scenario, the use of autophagy inducers has also been evaluated as potential cancer therapy alternatives [144,145,146,147].

The role of autophagy in the pathogenesis of human *cancer* appears to be complex, dynamic and has been associated with both tumor promoting and tumor suppressive mechanisms in a stage-dependent manner [148]. During the initial stages, autophagy represents a suppressive mechanism against tumor formation, counteracting the accumulation of damaged organelles and misfolded proteins and preventing genomic instability. In line with this, reduced expression levels or monoallelic deletions of BECN1 have been detected in human breast cancer, prostate, and ovarian cancer [149]. Similarly, defects of many other essential autophagy genes such as UVRAG, ATG5, ATG7 and ATG4C have been associated with higher risk of developing malignancies [150,151]. On the contrary, in later stages, autophagy could protect tumor cells in unfavorable stress conditions, providing a survival strategy for established tumor growth and maintenance. Indeed, RAS-driven lung and pancreatic cancers are more sensitive to acute loss of autophagy than most normal tissues and increased autophagic flux has been observed in various types of tumors [103,152]. In a clinical context, this biphasic role of autophagy in tumorigenesis needs to be taken into account *in order to* enable our prediction of the outcome of any potential autophagy-related therapies. Very recently Cassidy et al. demonstrated that systemic autophagy inhibition dramatically accelerated the aging process and induced premature acquisition of age-associated pathologies and a reduction in longevity in mouse models. Remarkably, autophagy restoration partially reversed ageing phenotypes, but autophagy-restored mice were associated with higher spontaneous tumor formation [153]. Although it may be strictly dependent on the extent and duration of treatment, these results suggest that the irreversible damage induced by autophagy inhibition might confer tumor susceptibility; afterwards, autophagy activity restoration can boost the malignant transformation and tumor maintenance, suggesting that the temporal use of autophagy inhibitors as cancer therapy could predispose patients to an increased long-term risk of secondary malignancies.

Resistance to chemotherapy represents a major clinical problem for the management of cancer patients. Although many lines of evidence suggest the feasibility of autophagy as a target in anticancer therapy, it remains unclear precisely what factors determine these opposing functions of autophagy, as results may be viewed as controversial so far. Although one possible explanation is that the functional consequences of autophagy may be different according to tissue of origin and stage of progression, there are likely to be as yet undefined bio-chemical and/or molecular factors that dictate the impact of autophagy on cell fate. Thus, the identification of specific tumor types and biomarkers that facilitate our understanding and rationale to either inhibit or activate autophagy as a therapeutic strategy in cancer is of great interest and is expected to produce novel, practical outputs of future benefit to patients with difficult-to-treat malignancies. 

## 11. Conclusions

Genomic instability is the main etiological factor for carcinogenesis and plays a critical role in neurodegenerative diseases and aging. Cells have evolved complex mechanisms to preserve genome integrity and, when environmental and endogenous DNA damage-inducing agents threaten genomic stability, DDR is activated, leading to the detection, signaling and repair of lesions or, if damage is irreparable, to senescence or cell death.

In 2007, autophagy was reported for the first time to protect genomes [154]. From then on, many roles have been proposed for autophagy in preventing genomic instability, as well as in participating in DDR and cell fate decision after DNA damage. Genomic stabilizing functions of autophagy include mitochondrial quality control and reduction in ROS, defense against cellular carcinogenic pathogens, degradation of overexpressed or misfolded proteins and engulfment of micronuclei and damaged nuclear parts. In addition to its protective role, autophagy can also influence the dynamics of DDR and the processing of genomic lesions. Significant evidences indicate that autophagy is closely integrated with the DDR network: DNA damage can induce autophagy under both physiological and pathological conditions and cells require proficient autophagic flux for proper activity of the DNA repair machinery. Moreover, autophagy ensures bio-energetic fitness providing metabolic precursors for the generation of ATP, required for optimal DNA repair, and maintains nucleotide homeostasis for DNA synthesis. However, in the case of excessive or prolonged DNA damage, autophagy ultimately facilitates senescence or cell death, thereby avoiding the proliferation of cells with genomic aberrations. 

The crosstalk between autophagy and DDR is just beginning to be explored and key questions remain that need to be addressed. The use of integrative omics and emerging biological systems provides a great opportunity to reach a full understanding of the complex and intricate relationship between autophagy and genome maintenance and could have profound impacts on our ability to treat cancer, age-related pathologies and neurodegeneration with autophagy manipulation.
